# Who Wants to Have an AI Therapist? Acceptance of Using Artificial Intelligence for Mental Health Interventions Among Clinicians, Patients and the General Community

**DOI:** 10.1002/cpp.70220

**Published:** 2026-01-23

**Authors:** Vera Békés, Katie Aafjes‐van Doorn

**Affiliations:** ^1^ Department of Educational and Counselling Psychology McGill University Montreal Canada; ^2^ Ferkauf Graduate School of Psychology Yeshiva University Bronx New York USA; ^3^ Arts and Sciences New York University Shanghai Shanghai China

**Keywords:** artificial intelligence, avatar, chatbot, digital mental health interventions, technology acceptance, teletherapy

## Abstract

The integration of AI‐based digital technologies in mental healthcare represents a transformative shift, especially with regard to chatbots and avatar‐based interventions. A central component of the success of AI‐based digital mental health interventions has to do with the level of acceptance of this new technology: the degree to which stakeholders perceive a technology as useful, user‐friendly and worth adopting. We aimed to establish the level of acceptance of AI‐based digital mental health interventions (AI chatbot, AI avatar‐based interventions) compared with the acceptance levels of teletherapy via videoconferencing among clinicians, patients and a representative community sample (i.e., potential future patients). We also explored the extent to which these differences towards these technologies might be explained by individuals' attitudes towards AI in general. Clinicians (*N* = 658), patients (*N* = 451) and US census–based community sample (*N* = 520) completed standardised measures of everyday artificial intelligence use, general attitudes towards AI and acceptability of digital technology use for mental health interventions. We found that community participants are most optimistic about AI‐based mental health tools (chatbots and avatars), whereas clinicians consistently express more scepticism, especially regarding usability. In our sample, general attitudes towards AI (both positive and negative) were highly associated with acceptance of chatbot and avatar‐based interventions, more than their professional role or demographic identity. These findings might carry clinical implications for the design, deployment and integration of these technologies into mental health services.

## Summary

- Across the samples, teletherapy was much more accepted than AI‐based interventions, and chatbots were slightly more accepted than AI Avatars.
- Community participants were most optimistic about AI‐based interventions (chatbots and avatars), and clinicians were most sceptical, whereas patients fell in between.
- Within this sample, general attitudes toward AI were strongly associated with acceptance of AI‐based mental health interventions.

The mental health field, amid a global crisis characterised by increasing demand and insufficient resources, is witnessing a significant paradigm shift facilitated by digital interventions. Digital mental health interventions (DMHIs) are mental health treatments delivered via digital technologies, including mobile apps, web‐based platforms, text messaging services, chatbots, teletherapy through videoconferencing (Mohr et al. 2017) and most recently, various artificial intelligence–based tools, such as chatbots and avatars. These DHMIs aim to increase accessibility to care, improve engagement and continuity and reduce barriers such as geographic distance or stigma (e.g., Shao 2023).

## 1 AI‐Based Interventions

In recent years, AI has begun to be implemented in multiple domains of mental health care (Graham et al. 2019). For example, AI‐based solutions have been used to improve the diagnosis of depression (Cicconet et al. 2022) and dementia (Chen et al. 2023) and to predict treatment outcomes (Chekroud et al. 2021). Intelligent robots have been shown to support children with autism spectrum disorders (e.g., Frenkel et al. 2025) and elderly people suffering from dementia (e.g., Góngora Alonso et al. 2019; Steijger et al. 2025). And virtual reality (VR) avatars have allowed psychotic patients to confront their auditory hallucinations (e.g., Craig et al. 2018; Sivaji et al. 2025).

### 1.1 Chatbots

The most commonly used AI application for DMHI is the mental health chatbot. Chatbots have become popular in healthcare in general and mental health specifically in the past 5 years. According to a scoping review conducted by Abd‐alrazaq et al. in 2019, there were already 41 different chatbots used for several purposes in mental health in 2018, such as therapy, training, education, counselling and screening. The number of available chatbots is constantly and rapidly growing. The global market for AI mental health chatbots was valued at approximately $1.38 billion in 2025.^1^ Patients' positive attitudes and engagement with chatbots, as well as positive mental health outcomes, show that chatbot technology is a promising modality for mental health intervention (see systematic reviews by Abd‐Alrazaq et al. 2020; Limpanopparat et al. 2024). Many of these chatbots used to be based on relatively simple dialogue systems (Laranjo et al. 2018); however, in recent years, these chatbots have become more human‐like and effective (Feng et al. 2022; Feng et al. 2025), and acceptable to patients (Ng and Zhang 2025) as well as health professionals (Baek et al. 2025).

Future enhancements are expected to introduce more natural, real‐time voice interactions and enable conversations with ChatGPT through real‐time video, broadening the potential applications of AI in DMHI. The upcoming GPT‐5 is anticipated to surpass the current capabilities of GPT‐4, further augmenting the effectiveness of AI applications in mental health care and extending the range of services available to patients.

### 1.2 AI Avatars

An AI avatar is defined as an embodied virtual agent that uses a machine learning model to process information to interact with humans in the mental health context. Research suggests that avatar‐based interventions have the potential to reduce barriers to therapy through anonymity, ease of access and potential for self‐disclosure, while allowing for meaningful nonverbal communication (Jang et al. 2025). Avatar‐based interventions appear to be acceptable to patients (Thompson et al. 2023). Thus far, the most common application of avatar‐based therapy is for the treatment of auditory hallucinations. Recent findings suggest that AVATAR therapy, in which the patient and therapist interact with a digital embodiment of patients' auditory hallucinations, effectively reduces distress and symptom frequency, which could be a game‐changer for patients (Nordentoft 2024).

Notably, most of the literature on AI‐DMHI so far reflects the tech‐savvy nature of scientists. Anecdotally, and supported by initial research, clinicians are generally less excited about these technological developments. For example, issues are raised about data privacy (Hasal et al. 2021), and professionals stress the importance of human empathy, understanding of complex emotional states and the ability to provide personalised, evidence‐based interventions that AI currently cannot fully replicate (e.g., Khawaja and Bélisle‐Pipon 2023). Clinicians argue that such tools cannot or should not replace human therapists (e.g., Brown and Halpern 2021). Patients appear to be somewhat more positive and open to using AI‐based tools if this helps them get better quality interventions (Aafjes‐van Doorn 2025). The results of a scoping review suggested that patients had overall positive opinions about the use of chatbots for mental health (Abd‐Alrazaq et al. 2021).

Despite the potential of AI applications in mental health, our current understanding of clinicians' and patients' perspectives of AI, especially AI avatar usage in mental health context, is limited (Moriuchi 2025). Even if AI‐DMHIs are technically feasible and are, or will be, of high quality according to developers, the clinical feasibility of the implementation itself will depend on the perspective of the clinicians and especially the (potential future) patients.

In other words, to ensure optimal utilisation of these tools, we need to understand stakeholders' view of these AI‐DMHIs: clinicians (who have the most expertise in how interventions actually work), patients (who had their own lived experience of interventions, regardless of the intervention format) and the community in general. Acceptability by individuals in the community is not only important as they might be future patients; they are likely also the main audience of AI‐DMHIs as they are mostly targeting individuals with mild symptoms and those who otherwise would not see a clinician (Vaidyam et al. 2019). Given the rapid development of advancements in AI technologies, it is only natural to wonder whether AI already is, or will be in the future, an acceptable tool for providing mental health interventions.

## 2 Teletherapy

Since the pandemic, teletherapy via videoconferencing has become widely practiced around the world and is now the most common method of providing mental health interventions (Olfson et al. 2025). In a short time, teletherapy via videoconferencing has become acceptable, even in traditionally less technology‐savvy populations, such as mature female therapists, as well as among practitioners of psychoanalysis, where the traditional frames play a central role in treatment (Aafjes‐van Doorn et al. 2022; Békés et al. 2020). For example, in a 2020 study, Békés and colleagues surveyed 1257 clinicians (77% female, mean age = 51 years) and found that many held concerns about the efficacy and relational aspects of teletherapy. However, therapists with more experience in teletherapy reported more positive attitudes, suggesting that familiarity may mitigate initial scepticism. Similarly, studies of patient populations (e.g., Feijt et al. 2020) report higher usage rates and satisfaction among women using teletherapy. Empirical research indicates that both gender and age significantly influence the acceptability of teletherapy via videoconferencing (e.g., Connolly et al. 2020). It appears that younger women are often the most receptive group: engaged, tech‐savvy and more open to therapy more generally. Older men tend to be the least receptive, often citing discomfort with both technology and help‐seeking (Lucas and Villarroel 2022).

## 3 Theoretical Framework for Acceptability of AI‐Based DMHIs

Like the recent developments in teletherapy via videoconferencing, the future adoption of AI‐DMHI technologies will be influenced by patients' and clinicians' attitudes towards these technologies. There are a few conceptual frameworks that have been developed around the AI applications in mental health. For example, Grodniewicz and Hohol's model (2023) focuses on challenges and outlines three key barriers to AI delivering effective psychotherapy: the problem of a confused therapist, the problem of a nonhuman therapist and the problem of a narrowly intelligent therapist. Another framework is the technology acceptance model (TAM; Davis 1989), which has been used to identify key factors that predict the acceptance of digital mental health tools (Park and Kim 2023), including perceived ease of use (i.e., whether using the technology is free of effort) and perceived usefulness (i.e., whether the technology will enhance one's performance) (Davis 1989). This is important given that behavioural intent has long been recognised as an effective proxy measure of actual behaviour (Davis 1989).

The Unified Theory of Acceptance and Use of Technology (UTAUT) is built on the integration of previous models, including the TAM, and is presently the most comprehensive and most used model. Originally proposed by Venkatesh and colleagues (Venkatesh et al. 2003; Venkatesh et al. 2016), the UTAUT conceptualises acceptance of new technologies in professional settings and identifies determinants of behavioural intention to use a given technology. The UTAUT model expands previous models by incorporating social and environmental facilitating conditions and is particularly useful for understanding various individual‐level factors of technology acceptance. These factors are essential in the early stages of technology adoption, as they directly affect whether individuals will engage with and continue to use a technology. Indeed, a meta‐analysis conducted in 2023 (Li et al. 2023) that investigated the determinants that impact users' usage intention of AI‐based chatbots suggests that attitude, perceived usefulness and trust are critical factors for the adoption of AI‐based chatbots. They also reported that gender had a moderating role between the relationship of attitudes and user intention.

## 4 Aims

In our present study, we aimed to establish the level of acceptance of using AI DMHIs among main stakeholders: clinicians, patients and a representative community sample (i.e., potential future patients). Specifically, we sought to answer the following research questions:

1. What is the level of acceptance of three types of DMHIs: AI‐chatbot, AI avatar‐based intervention and teletherapy via videoconferencing among clinicians, patients and the general community?
2. What are the UTAUT factors relevant to acceptance of these three AI‐based DMHIs (AI chatbot, AI avatar and teletherapy) in the three samples (clinicians, patients and the general community)?
3. Can the group differences between clinicians, patients and the general community in the acceptance of the AI‐DMHIs (AI chatbot and AI avatar) be explained by their attitudes towards AI in general?

## 1 Methods

### 1.1 Procedures

The study was preregistered on the Open Science Framework (OSF) portal, at https://osf.io/qz48m. We recruited participants via Prolific, an academic crowdsourcing platform, established as best performing among crowdsourcing platforms (Albert and Smilek 2023; Douglas et al. 2023). We chose this platform to gather high quality, valid data in a concise time frame, based on clear inclusion and exclusion criteria, and to avoid usual pitfalls associated with recruitment over mailing lists and social media platforms, such as selection bias and invalid chatbot responses.

To collect Clinician and Patient samples, we used Prolific's preset screening questions as well as further screening questions built in the survey to identify participants in the clinician and patient groups. For patients, we selected participants who were currently receiving or waiting for psychological therapy or counselling for a mental health condition. For clinicians, we selected participants who identified as at least one of the following: clinician, mental health counsellor, providing mental health interventions (including assessment, psychotherapy, counselling) to patients either at present or in the past, working or having worked in the mental health field with at least 1 year of clinical experience. The patients were recruited from Prolific participants residing in the United States, and the therapists were recruited from the United States, United Kingdom and Canada. Finally, the community sample included a representative sample based on US census data on sex, age and ethnicity in Prolific, without any further screener questions.

Eligible participants received a web link with additional study details. Participants completed survey questions in a fixed order. The total survey took approximately 30 min to complete. Responses were forced for all questions. To ensure high data quality, various checks, including attention and bot detection measures, were implemented. Those participants who completed at least the UTAUT‐AI‐DMHI, the main outcome measure, were included in the present study. A previous study reported on a subsample of this data and on predictors of clinicians' acceptance of AI for mental health interventions (Békés and Aafjes‐van Doorn 2026).

Data collection occurred in several batches between 15 July 2024 and 13 January 2025. Participants were compensated via Prolific. The study was reviewed and deemed exempt by Yeshiva University's Institutional Review Board (#1‐1758995‐1), and all participants provided informed consent. The data used in this study are available from the corresponding author upon reasonable request.

### 1.2 Participants

The clinician group consisted of *N* = 658 participants with an average age of 37 years (SD = 10.97). Most clinicians identified as White (*N* = 378, 57.4%), women (*N* = 378; 57.4%) and from the United States (*N* = 452; 68.7%). Most clinicians had at least an undergraduate degree (*N* = 589, 89.5%), worked full or part‐time (*N* = 626, 95.1%) and had on average 5.27 (SD = 4.6) years of clinical experience. Most clinicians identified as clinicians or mental health counsellors (*N* = 473, 71.9%) and reported that they provided mental health interventions at present (*N* = 341, 61.8%).

The patient group included *N* = 451 participants with an average age of 38 years (SD = 11.35). Most patients identified as White (*N* = 328, 72.7%), women (*N* = 260; 57.6%) and from the United States (*N* = 435; 96.5%). Most patients had at least an undergraduate degree (*N* = 263; 58.3%) and worked full or part‐time (*N* = 312, 69.2%). Most patients were in therapy (*N* = 398, 88.2%), and a small minority was waiting for treatment (*N* = 53, 8.51%).

The US census–based community sample consisted of *N* = 520 participants with an average age of 46 years (SD = 16.17), identified as White (*N* = 375, 72.1%), with about half of them being women (*N* = 270; 51.09%). All community participants were from the United Sates; most of them had at least an undergraduate degree (*N* = 322, 61.9%) and worked full or part‐time (*N* = 335, 64.4%). For detailed data about demographics and professional characteristics of the study sample, see Table 1.

**TABLE 1:** Descriptive statistics of clinicians, patients, and representative community sample.

	Clinicians (*n* = 658)	Patients (*n* = 451)	Community sample (*n* = 520)
Age M (SD)	36.72 (10.97)	38.09 (11.35)	45.89 (16.17)
	*N* (%)	*N* (%)	*N* (%)
Gender			
Female	378 (57.4%)	260 (57.6%)	270 (51.09%)
Male	267 (40.6%)	162 (35.9%)	247 (47.5%)
Nonbinary/other	13 (2.0%)	29 (6.4%)	3 (0.6%)
Ethnicity*			
White European, European American	387 (58.8%)	328 (72.7%)	375 (72.1%)
Asian or Asian Indian	57 (8.7%)	19 (4.2%)	11 (2.1%)
Hispanic, Latinx, Spanish	42 (6.4&)	38 (8.4%)	53 (10.2%)
Black or African American	158 (24.0%)	87 (19.3%)	76 (14.6%)
American Indian or Alaska Native	9 (1.4%)	19 (4.2%)	7 (1.6%)
Middle Eastern	12 (1.8%)	5 (1.1%)	5 (1.0%)
Other	14 (2.3%)	9 (1.3%)	11 (2.1%)
Country			
United States	452 (68.7%)	435 (96.5%)	520 (100.0%)
Canada	22 (3.3%)	4 (0.9%)	
Europe	56 (12.4%)	4 (0.9%)	
United Kingdom	115 (17.0%)	3 (0.7%)	
Asia	4 (0.01%)	2 (0.4%)	
Australia	1 (0.00%)	3 (0.7%)	
Highest degree			
Less than high school	1 (0.2%)	6 (1.3%)	1 (0.2%)
High school or G.E.D.	17 (2.6%)	110 (24.4%)	118 (22.7%)
Associate's degree	42 (6.4%)	62 (13.7%)	74 (14.2%)
Undergraduate (college/university)	210 (31.9%)	161 (35.7%)	198 (38.1%)
Master's	306 (46.5%)	84 (18.6%)	103 (19.8%)
Doctoral (PhD, PsyD)	57 (8.7%)	11 (2.4%)	9 (1.7%)
Professional (MD, JD)	16 (2.4%)	7 (1.6%)	12 (2.3%)
Other	9 (1.4%)	10 (2.2%)	5 (1.0%)
Employment status*			
Works full or part time	626 (95.1%)	312 (69.2%)	335 (64.4%)
Does not work at the moment	10 (1.5%)	58 (12.9%)	54 (10.4%)
Retired	3 (0.5%)	11 (2.4%)	70 (13.5%)
Student	38 (5.8%)	37 (8.2%)	41 (7.9%)
Disability prevents them from working	3 (0.5%)	46 (810.2%)	12 (2.3%)
Has unpaid work in or outside the house	7 (1.1%)	20 (4.4%)	22 (4.2%)
Other	3 (0.5%)	10 (2.2%)	5 (1.2%)

*Multiple responses were allowed.

### 1.3 Measures

#### 1.3.1 Everyday Artificial Intelligence Use

We assessed regular AI use with the Daily Smart Technology Use measure by Bergdahl et al. (2023). Participants were given the following instruction: ‘Which of the following do you use regularly (i.e., at least once a week)?’ Participants were asked to give binary responses (yes/no). The listed items included four of the original items from Bergdahl et al. (2023): (1) a mobile robot or another intelligent device (e.g., robot vacuum cleaner, robot lawn mower, assistance robot); (2) a virtual assistant via smart speaker, computer or smartphone app (e.g., Siri, Alexa) and wearable smart technology (e.g., smart watch, smart ring); (3) augmented reality (AR) technology and (4) VR technology. We added four additional items: (5) Chat GPT, Bing AI and Baidu Ernie; (6) social media (e.g., X/Twitter, Instagram); (7) mental health app (e.g., meditation, sleep, such as Headspace); (8) mental health chatbot (e.g., Wysa, Wombat, Ada Health). A sum of these eight types of smart technologies was used to represent participants' daily AI use in our data analysis.

#### 1.3.2 General Attitudes Towards AI

The General Attitudes towards Artificial Intelligence Scale (GAAIS; Bergdahl et al. 2023), a shortened version of the original 20‐item GAAIS (Schepman and Rodway 2020, 2023), was used to assess attitudes towards AI in general. The short GAAIS consists of four positively framed items (e.g., ‘Much of society will benefit from a future full of Artificial Intelligence’, ‘Artificial Intelligence can provide new economic opportunities for this country’) and four negatively framed items (e.g., ‘I find Artificial Intelligence sinister’, ‘Artificial Intelligence might take control of people’.) Items are rated on a 5‐point Likert scale from 1 (*strongly disagree*) to 5 (*strongly agree*). Separate mean scores for the positive and negative items were calculated to derive positive and negative general attitude scores. The Cronbach's alpha was 0.88 for the positive subscale and 0.82 for the negative subscale in our study.

#### 1.3.3 Acceptability of Using Digital Technology for Mental Health Interventions

**FIGURE 1:**
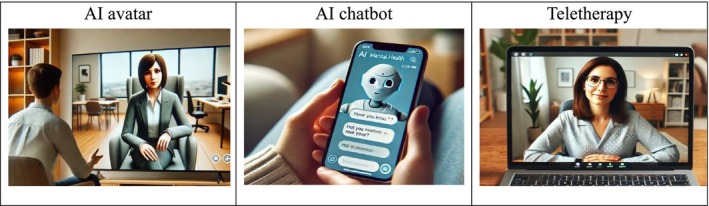
Illustration of the instructions for completing the UTAUT‐AI‐DMHI*.* *Note:* Figure initially reported in Békés et al. (2025). Instruction: ‘Traditionally, mental health counselling and psychotherapy have been provided by human therapists. Recently, there has also been the option of working on mental health concerns with an AI‐based therapist. Unlike human therapists via teleconferencing, AI‐based therapists are available anytime 24/7. AI‐based therapists could take two forms: 1) AI chatbot who interacts with you via text‐messaging; most people report that it's as if they are texting with a human therapist. 2) AI virtual therapist avatar who you can see, hear and talk to via a computer screen; the AI virtual therapist is trained to perform therapy as a human therapist and looks and speaks like a human’. *AI‐generated images by ChatGPT DALL·E (OpenAI 2024). The original teletherapy image included a human therapist.

The UTAUT Artificial Intelligence and DMHI (UTAUT‐AI‐DMHI; Békés et al. 2025) was used to evaluate acceptance of (1) teletherapy via video conferencing by a human clinician, (2) AI chatbot and (3) AI virtual clinician (avatar) interventions. The UTAUT‐AI‐DMHI consists of 17 items, along with an additional item measuring behavioural intention to use the specific DMHI in the future. The scale has seven subscales: ease of use, social influence, convenience, human connection, perceived privacy risk, hedonic motivation and therapy quality expectations, plus the one‐item asking about future intention of utilisation of the given technology. Participants responded to the same set of items three times, once for each digital technology (teletherapy, AI chatbot, AI avatar) (see Figure 1). Each item was rated on a 5‐point Likert scale ranging from (1) strongly disagree to (5) strongly agree. To support participants during completion, descriptions and visual aids illustrating the three types of therapies were provided (see Figure 1). The UTAUT‐AI‐DMHI demonstrated strong psychometric properties: The entire scale and its factors showed adequate construct validity, reliability and concurrent validity, and each factor was positively associated with general attitudes towards AI and intention to use the assessed DMHI format in the future (Békés et al. 2025). The alpha in our study was 0.83 for teletherapy, 0.87 for the AI chatbot and 0.89 for the AI virtual clinician.

### 1.4 Data Analysis

No missing data were imputed as we only included participants who provided data on the main outcome measure. Preliminary analyses included bivariate zero‐order Pearson's correlations to establish relationships between the study variables. Levene's test was used to indicate whether the assumption of homogeneity of variances was met. Due to the low number of non‐gender binary participants (*N* = 45, 2.88%), reported gender was treated as a binary variable. To examine whether the three groups differed significantly in age, a one‐way ANOVA was conducted, with group (clinician, patient, community) as the independent variable and age as the dependent variable. Post hoc comparisons were performed using Tukey's test to identify which group differences were statistically significant. To compare the groups for gender and environment (urban versus other), chi‐square tests of independence were conducted.

For Research Questions 1 and 2, that is, to establish and compare acceptance of AI‐based mental health interventions and identify the UTAUT factors that may be linked to these differences, we conducted one‐way ANOVA comparing group differences (clinician, patient, community) on UTAUT‐AI‐DMHI for chatbot, avatar and teletherapy mean scores and subscale scores and established specific group differences using Tukey post hoc test. To assess the potential confounding effects of age and gender, we conducted ANCOVAs including these variables as covariates. In cases where the inclusion of covariates attenuated or eliminated significant group differences, we conducted matched‐sample analyses to further explore the role of these demographic variables. If relevant, we planned to use paired‐samples *t*‐tests to compare matched subsamples on the outcome variables, thereby testing whether group differences persisted when demographic variables were held constant. To examine Research Question 3 and establish whether the group differences in AI chatbot and AI avatar are maintained even after controlling for positive and negative attitudes towards AI in general, we used ANCOVA with positive and negative general AI attitudes as covariates. Finally, post hoc we also tested whether there was an order effect in the three DMHI modalities acceptance scores by conducting a repeated‐measures ANOVA with presentation order as within‐person factor to check for any polynomial effect.

## 2 Results

### 2.1 Preliminary Analysis

Age was significantly associated with the total acceptance scores on the UTAUT‐AI‐DMHI. Specifically, older age was associated with more positive attitudes towards AI chatbot and AI avatar, but age was unrelated to teletherapy acceptance, see details in Table 2. There were significant gender differences in that male participants reported lower levels of acceptance of teletherapy (*t*_teletherapy_ = 0.09, *p* < 0.001) and more positive views towards AI chatbot (*t*_chatbot_ = −7.07, *p* < 0.001) and AI avatar (*t*_avatar_ = −8.67, *p* < 0.001). Moreover, there were significant differences in UTAUT scores for participants living in an urban (*N* = 657) versus other environments (*N* = 994) (*t*_teletherapy_ = −0.145, *p* = 0.884, *t*_chatbot_ = 1.21, *p* = 0.227; *t*_avatar_ = 1.36, *p* = 0.176).

**TABLE 2:** Zero‐order correlations between the study variables.

	Age	Chatbot	Avatar	Teletherapy	GAAIS	GAAIS
					pos	neg
Age	—					
Chatbot	0.13**	—				
Avatar	0.17**	0.85**	—			
Teletherapy	−0.02	0.00	0.01	—		
GAAIS pos	0.11**	0.44**	0.47**	0.17**	—	
GAAIS neg	−0.12**	−0.36**	−0.39**	−0.12**	−0.50**	—

*Note:* Teletherapy = UTAUT‐AI‐DMHI for Teletherapy. Chatbot = UTAUT‐AI‐DMHI for Chatbot. Avatar = UTAUT‐AI‐DMHI for Avatar. UTAUT‐AI‐DMHI. GAAIS pos = General Attitudes towards Artificial Intelligence Scale (GAAIS; Bergdahl et al. 2023), mean of positive items; GAAIS neg and GAAIS pos = Negative and Positive General Attitudes towards Artificial Intelligence Scale (GAAIS; Bergdahl et al. 2023).

** *p* < 0.001.

Levene's test for homogeneity of variances for the UTAUT‐AI‐DMHI was not significant, *F*(2, 1626) = 0.780, *p* = 0.459, indicating that the assumption of equal variances was met. One‐way ANOVAs showed a significant effect of group on age and gender, *F*(2, 1672) = 82.08, *p* < 0.001, and *F*(2, 1626) = 5.14, *p* = 0.006, respectively. Post hoc comparisons using Tukey's test indicated that the average age of the community sample (*M* = 45.89, SD = 16.17) was significantly higher than the sample of clinicians (*M* = 36.67, SD = 10.94, *p* < 0.001) and patients (*M* = 38.06, SD = 11.43, *p* < 0.001). A chi‐square test indicated that gender distribution differed significantly across the three samples, *χ* ^2^(2, *N* = 1629) = 10.23, *p* = 0.006. A smaller percentage of the community participants were female (*N* = 270; 51.9%), compared with the percentage of females among the other two groups: clinicians (*N* = 378; 57.4%) and patients (*N* = 260, 57.6%). No significant age or gender difference was found between patients and clinician participants (*p* = 0.172, and *p* = 0.065, respectively).

### 2.2 Research Question 1: What Is the Level of Acceptance of Digital Mental Health Interventions Among Clinicians, Patients and the Community?

In the overall sample of *N* = 1629, acceptance for AI chatbot was 2.92 (SD = 0.80), for AI avatar 2.81 (SD = 0.77), both reflecting neutral to slightly negative views (3 referring to ‘neutral’ and 2 to ‘disagree’ with the scale items on a 5‐point scale). Acceptance of teletherapy was *M* = 3.81 (SD = 0.59) reflecting reasonably positive views (4 referring to agree with the scale items). Repeated measures ANOVA showed that these differences between the three formats were statistically significant, *F*(2, 1627) = 863.36, *p* < 0.001; specifically, teletherapy was rated significantly higher than both the AI Avatar, *t*(1628) = −41.51, *p* < 0.001, and the Chatbot, *t*(1628) = −36.54, *p* < 0.001. The AI chatbot was also rated higher than the AI Avatar, *t*(1628) = 8.86, *p* < 0.001.

One‐way ANOVAs comparing the clinician, patient and community samples on acceptance of AI chatbot, AI avatar and teletherapy indicated significant group differences, *F*(2, 1626) = 2.34, *p* = 0.036, and *F*(2, 1626) = 3.23, *p* = 0.004, *F*(2, 1626) = 19.65, *p* < 0.001, respectively. Tukey post hoc test indicated that the community sample had significantly more acceptance of AI chatbots and AI avatars compared with both the clinician and patient groups, whereas clinicians and patients did not differ significantly.

**FIGURE 2:**
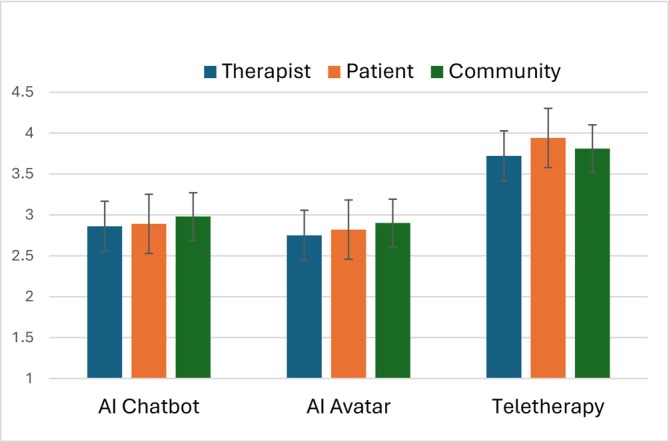
Acceptance of digital mental health interventions (*N* = 1629). *Note:* DMHI, digital mental health interventions. Possible range of the UTAUT‐DMHI‐AI total score 1 (*totally disagree*, i.e., negative attitude) to 5 (*totally agree*, i.e., positive attitude).

Acceptance of teletherapy was highest among patients, then in the community samples, and the lowest among clinicians. According to the Tukey test, the difference between each group was significant for teletherapy acceptance (see Figure 2 and Table 3).

**TABLE 3:** Means, standard deviations, and differences across the clinician, patient, and community samples on attitudes towards using AI chatbots, AI avatars, and teletherapy for mental health interventions.

UTAUT‐AI‐DMHI	Clinician	Patient	Community	*F*	*p*	*η* ^2^	T vs. P p	P vs. C p	T vs. C p
AI chatbot subscales	*M* (SD)	*M* (SD)	*M* (SD)						
Therapy quality exp.	2.45 (1.07)	2.43 (1.06)	2.57 (1.06)	2.53	0.080	0.003	0.943	0.106	0.145
Ease of use	3.11 (1.02)	3.35 (1.04)	3.35 (1.05)	10.56	**< 0.001**	0.013	**< 0.001**	0.998	**< 0.001**
Social influence	3.82 (1.70)	3.70 (1.52)	3.88 (1.51)	1.45	0.231	0.002	0.461	0.210	0.811
Convenience	3.50 (1.11)	3.82 (1.07)	3.77 (1.13)	13.83	**< 0.001**	0.017	**< 0.001**	0.691	**< 0.001**
Human connection	2.06 (0.97)	2.00 (0.96)	2.17 (1.00)	3.03	0.**020**	0.005	0.531	0.**017**	0.142
Perc. privacy risk	2.34 (1.79)	2.30 (1.28)	2.43 (1.23)	1.36	0.257	0.002	0.844	0.248	0.470
Hedonic motivation	2.72 (1.11)	2.59 (1.07)	2.70 (1.09)	1.92	0.147	0.002	0.141	0.299	0.936
Behavioural intention	2.53 (1.41)	2.66 (1.36)	2.71 (1.37)	2.72	0.132	0.002	0.290	0.835	0.073
Overall score	2.86 (0.80)	2.89 (0.77)	2.98 (0.79)	3.34	0.**036**	0.004	0.758	0.224	0.**029**

UTAUT‐AI‐DMHI	Clinician	Patient	Community	*F*	*p*	*η* ^2^	T vs. P p	P vs. C p	T vs. C p
AI avatar subscales	*M* (SD)	*M* (SD)	*M* (SD)						
Therapy quality exp.	2.70 (1.13)	2.68 (1.11)	2.83 (1.09)	2.75	0.061	0.003	0.941	0.085	0.118
Ease of use	3.18 (0.98)	3.38 (0.98)	3.42 (0.96)	10.26	**< 0.001**	0.012	0.**002**	0.773	**< 0.001**
Social influence	2.72 (1.71)	2.66 (1.04)	2.79 (1.02)	1.89	0.151	0.002	0.600	0.128	0.502
Convenience	3.51 (1.04)	3.86 (0.99)	3.86 (1.01)	23.65	**< 0.001**	0.028	**< 0.001**	0.999	**< 0.001**
Human connection	2.01 (0.92)	2.00 (0.96)	2.15 (0.97)	4.12	0.**016**	0.005	0.987	0.**038**	0.**031**
Perc. privacy risk	2.25 (1.15)	2.32 (1.25)	2.40 (1.22)	2.08	0.125	0.003	0.601	0.613	0.104
Hedonic motivation	2.82 (1.13)	2.72 (1.08)	2.80 (1.09)	1.26	0.285	0.002	0.274	0.459	0.953
Behavioural intention	2.75 (1.48)	2.76 (1.38)	3.81 (1.38)	0.281	0.755	0.000	0.980	0.875	0.741
Overall score	2.75 (0.77)	2.82 (0.75)	2.90 (0.76)	5.54	0.**004**	0.007	0.301	0.233	0.**003**

UTAUT‐AI‐DMHI	Clinician	Patient	Community	*F*	*p*	*η* ^2^	T vs. P p	P vs. C p	T vs. C p
Teletherapy subscales	M (SD)	M (SD)	M (SD)						
Therapy quality exp.	4.16 (0.73)	4.30 (0.76)	4.16 (0.74)	5.88	0.**003**	0.007	0.**006**	0.**008**	0.998
Ease of use	3.75 (0.83)	3.97 (0.84)	3.99 (0.79)	16.22	**< 0.001**	0.019	**< 0.001**	0.916	**< 0.001**
Social influence	4.01 (0.87)	4.24 (0.79)	4.02 (0.86)	11.59	**< 0.001**	0.014	**< 0.001**	0.**001**	0.958
Convenience	4.08 (0.87)	4.19 (1.01)	4.09 (0.96)	2.22	0.109	0.003	0.116	0.207	0.977
Human connection	3.33 (1.05)	3.75 (1.03)	3.56 (0.99)	23.46	**< 0.001**	0.028	**< 0.001**	0.**012**	**< 0.001**
Perc. privacy risk	3.33 (1.19)	3.75 (1.17)	3.48 (1.18)	17.28	**< 0.001**	0.021	**< 0.001**	0.**001**	0.063
Hedonic motivation	3.39 (0.82)	3.39 (0.89)	3.40 (0.83)	0.029	0.971	0.000	0.998	0.986	0.969
Behavioural intention	4.25 (0.95)	4.36 (0.95)	3.95 (1.10)	3.64	0.057	0.003	0.176	**< 0.001**	**< 0.001**
Overall score	3.72 (0.56)	3.94 (0.62)	3.81 (0.57)	19.65	**< 0.001**	0.024	**< 0.001**	0.**002**	0.**013**

*Note:* Significant differences are bolded (*p* < 0.05).

Abbreviations: C, community sample; P, patients; perc privacy risk, perceived privacy risk; T, clinicians; Therapy quality exp., expectations of the quality of therapy; UTAUT‐AI‐DMHI = Unified Theory of Acceptance and Use of Technology Artificial Intelligence and Digital Mental Health Interventions (Békés et al. 2025).

Since age and gender composition was significantly different in the census‐based community sample compared with the clinician and patient samples, we conducted ANCOVAs to control for age and gender. When we included gender as a covariate, group differences remained significant; however, when we included age, group differences in acceptance of AI chatbot and AI avatar lost significance but remained significant in acceptance of teletherapy. However, because age was not independent from group membership (the community sample was older on average), it is possible that the ANCOVA overcorrected for age‐related effects, which could be in fact part of the group membership effect. This means that controlling for age may have removed variance that in fact differentiated the groups. To better understand if group differences in DMHI acceptance hold after age difference between groups were removed, we matched the ages across the three groups and repeated the analyses. Specifically, participants in the clinician, patient and community groups were matched on age based on nearest‐neighbour matching. The analyses were then repeated using this age‐matched sample. This analysis still showed significant group differences, suggesting that age may have acted as a confounding variable in the full sample.

### 2.3 Research Question 2: What Are the UTAUT Factors That Underlie Differences in DMHI Acceptance?

For AI chatbots, clinicians perceived ease of use and convenience significantly less favourable compared with patients and the community sample, whereas the community sample viewed the possibility of Human connection more positively when using an AI chatbot compared to clinicians and patients.

For AI Avatar, clinicians perceived ease of use and convenience significantly less favourably compared with patients and the community sample, whereas the community sample viewed the possibility of human connection with an AI avatar significantly more positively compared with patients and clinicians.

Finally, for teletherapy, patients had significantly higher quality expectation and perceived a stronger social influence to use it compared with clinicians and the community sample, and patients perceived significantly more privacy risk in teletherapy compared with the other groups as well. Clinicians rated ease of use significantly lower than the other two groups. The three groups significantly differed in perceived human connection in teletherapy, with patients perceiving the highest connection, community sample the next and clinicians the weakest connection. Unsurprisingly, patients and clinicians were significantly more likely to use teletherapy in the future compared with the community sample. For details, see Table 3.

### 2.4 Research Question 3: What Is the Impact of General AI Attitudes on the Differences in Acceptance of Using AI for Mental Health Interventions?

We conducted ANCOVA analyses to establish whether group differences in DMHIs remain significant if we control for general AI attitudes. We conducted these analyses for AI chatbot and AI avatar interventions, as pertaining to AI directly. Results showed that positive general AI attitudes were significantly related to the acceptance of AI chatbots, *F*(1, 1629) = 373.26, *p* < 0.001, partial *η* ^2^ = 0.187. The effect of group (clinician, patient and community) on acceptance of AI chatbots was no longer significant once controlling for positive general AI attitudes, *F*(2, 1629) = 1.49, *p* = 0.22, partial *η* ^2^ = 0.002. Negative general AI attitudes were also significantly related to the acceptance of AI chatbots, *F*(1, 1629) = 234.91, *p* < 0.001, partial *η* ^2^ = 0.126. After controlling for negative general AI attitudes, the effect of group (clinician, patient and community) remained significant, *F*(2, 1629) = 3.54, *p* = 0.029, partial *η* ^2^ = 0.004.

In the case of AI avatar, positive general AI attitudes were significantly related to the acceptance of AI avatar, *F*(1, 1629) = 435.51, *p* < 0.001, partial *η* ^2^ = 0.211. After controlling for positive general AI attitudes, the effect of group (clinician, patient and community) on acceptance of AI avatar interventions remained significant, *F*(2, 1629) = 3.10, *p* = 0.045, partial *η* ^2^ = 0.004. Negative general AI attitudes were also significantly related to the acceptance of AI avatar, *F*(1, 1629) = 286.51, *p* < 0.001, partial *η* ^2^ = 0.150. After controlling for negative general AI attitudes, the effect of group (clinician, patient and community) on acceptance of AI avatar remained significant, *F*(2, 1629) = 5.19, *p* = 0.006, partial *η* ^2^ = 0.006. Finally, post hoc analysis of potential order‐effect showed significant linear and quadratic within‐person contrasts; however, these were driven by the large jump at the third modality (teletherapy) rather than order‐based trend between AI Avatar, AI chatbot and Teletherapy.

## 3 Discussion

Consistent with our aims, this study examined differences in acceptance of AI‐based mental health interventions across key stakeholder groups and assessed whether these differences were accounted for by general attitudes towards AI. Specifically, we sought to establish the level of acceptance of using three types of DMHIs: AI chatbot, AI avatar‐based intervention and teletherapy via videoconferencing among a sample of clinicians, patients and a representative community sample (i.e., potential future patients). To answer our research questions, we conducted three parallel survey studies in a sample of mental health clinicians (*N* = 658), patients (*N* = 451) and a US census‐based community sample (*N* = 520).

Overall acceptance of AI‐based DMHIs was neutral to slightly negative, whereas teletherapy was viewed more positively across groups. Community participants showed the greatest optimism towards AI‐based tools (chatbots and avatars), while clinicians expressed more scepticism, particularly regarding usability. Patients show the highest engagement and trust in teletherapy but are more concerned about privacy. As expected, general attitudes towards AI (both positive and negative) were highly associated with acceptance of chatbot and avatar interventions in our sample, more than professional role or demographic identity.

Acceptance of AI‐based interventions in our study mirrors the moderate acceptance levels seen for teletherapy early in the COVID‐19 pandemic, suggesting a similar trajectory of cautious openness that may increase with familiarity. At that time, patients (UTAUT‐P mean = 3.49, SD = 0.58) reported somewhat more positive attitudes towards teletherapy than therapists (UTAUT‐T mean = 3.42, SD = 0.51) (Békés and Aafjes‐van Doorn 2020; Békés et al. 2022). Notably, the acceptance levels of teletherapy have increased over time and with more use (Békés and Aafjes‐van Doorn 2020; Békés et al. 2022), a trend that may possibly also occur for the AI‐based interventions as people get more familiar with these technologies.

### 3.1 Acceptability of DMHIs

We found significant population differences in acceptance of all three types of DMHI. For AI‐chatbots and AI‐avatars, the community sample reported higher levels of acceptance than clinicians and patients. For teletherapy, patients were more positive than the community sample, who were more positive than clinicians. When controlling for age, differences in acceptability of AI chatbots and avatars disappeared, but differences in acceptability of teletherapy remained. When examining this further, a matched‐age analysis showed that age (the relatively higher age of participants in the community sample) may have at least partially confounded acceptance of AI‐based tools. This implies that controlling for age may have removed variance explained by other age‐related group differences; that is, the effect of age on more AI chatbot and avatar acceptance might be explained by the fact that there were more older males in the community sample (and vice versa, the effect of group might be at least partly related to between‐group age difference). This may mean that the community's favourable opinions about AI more generally and AI‐DMHIs specifically may be more important than their age per se.

Interestingly, older participants reported somewhat higher acceptance of AI chatbots and avatars, which challenges the assumption that older age is necessarily linked to lower technology acceptance. The age‐related differences may reflect other unmeasured factors rather than age per se, such as greater experience navigating barriers to health care or differing attitudes towards anonymity in mental health treatment, although these possibilities remain speculative given the cross‐sectional design.

Arguably, the fact that the community sample reported the most positive levels of acceptance towards AI‐based mental health interventions (chatbot and avatar) is promising, as these tools may have the potential to address stigma and improve access for individuals who might otherwise be reluctant to engage with psychological therapy, although this implication cannot be directly tested within the present data. The fact that clinicians had more negative views of the ease of use and convenience of AI chatbots or AI avatars is consistent with research suggesting greater technology scepticism among clinicians (Cecil et al. 2025), although such attitudes may also reflect professional norms and clinical experience rather than general technology pessimism per se. In contrast, community participants were more favourable about the ability to experience a human connection with an AI chatbot or AI avatar, possibly reflecting greater general optimism towards technology (Guingrich and Graziano 2025), differences in expectations about therapeutic relationships or limited comparative experience with in‐person or teletherapy. This would fit with the findings that clinicians and patients tended to be more likely to use teletherapy in the future than the community participants.

### 3.2 The Role of Attitudes Towards AI in General

In our sample, participants' general attitudes towards AI were strongly associated with acceptance levels of AI‐chatbots and AI avatars, with more positive attitudes related to higher acceptability and more negative attitudes related to lower levels of acceptability towards these AI‐DMHIs. That said, there were still significant differences between the three groups of participants, in that the community individuals who were more negative about AI generally were still more positive about AI via chatbots and AI avatars than the clinicians and patients. Similarly, the community individuals who viewed AI more positive generally reported significantly higher levels of acceptance towards AI avatars in DMHIs. Our sample findings underscore that general AI attitudes (both positive and negative) are strongly associated with acceptance of AI‐based DMHIs and may be as influential as professional role within this attitudinal context. This aligns with recent work showing that individual differences in technological affinity influence openness to AI in clinical contexts (Wagner and Schwind 2025). Future implementation strategies could therefore target these broader attitudes rather than focusing solely on demographics. Together, these findings highlight that professional background and general AI attitudes jointly shape acceptance; clinicians' scepticism may reflect lower technological confidence, whereas community participants' optimism may reflect broader enthusiasm for AI applications.

### 3.3 Limitations

Several limitations and future directions can be highlighted. First, our study design itself is limited in some ways. For example, because our analyses are cross‐sectional, all associations should be interpreted as correlational, and hypothesis‐generating rather than causal. Future longitudinal or experimental studies are needed to address questions of causal mechanisms. Another aspect of the study design regards our choice to compare two types of AI‐based interventions (chatbot and avatars) with teletherapy via videoconferencing. However, we did not compare individuals' acceptance level with in‐person therapy, which is still a common way in which psychotherapy is currently practiced.

Secondly, participants responded to the same set of items three times, once for each digital technology (teletherapy, AI chatbot, AI avatar). Even though we ruled out order‐based effects in responses, we used the same order of items for all participants, which might have possibly impacted their responses in the same way; for example, there may be even larger differences in scores if teletherapy with a human therapist was provided as the first option. Furthermore, adding in‐person therapy with a human therapist might have shifted the responses even more, when teletherapy, rated the highest in our study, could already be seen as a less satisfying option.

Third, our sample was mainly from the United States, which means that it is unsure how our results might generalise to other non‐western countries, such as China. In Asia, where in‐person mental health care is less established and mental health challenges are exacerbated by a shortage of professionals and cultural stigma (Bhat et al. 2025), these AI‐DMHIs offer scalable solutions and might thus be perceived more positively by clinicians and (future) patients. Indeed, it appears that young adults who are reluctant to engage with human‐delivered psychotherapy due to help‐seeking self‐stigma may be more inclined to seek help through alternative modes of psychotherapy, such as AI chatbots (Hoffman et al. 2024).

Fourth, the predictive performance indices were high (near‐ceiling ROC–AUC values, large odds ratios for individual predictors and high sensitivity and specificity estimates in the logistic regression and ROC analyses). These results demonstrate strong internal discrimination within the present sample, but do not necessarily suggest wider clinical prediction. The magnitude of the observed effects may partially reflect construct proximity between predictors and outcomes, as well as shared method variance due to reliance on self‐report measures. Thus, the present analyses are best interpreted as explanatory models of acceptance within this dataset, rather than as validated tools for individual‐level prediction or clinical decision‐making. Future work should evaluate whether comparable predictive performances are observed in independent samples and across alternative measurement modalities.

Also, general attitudes towards AI are conceptually adjacent to the outcome measures assessing acceptance and willingness to engage with AI‐based DMHIs. Although these constructs are theoretically distinguishable and were operationalised using separate scales, some degree of conceptual overlap is likely. This proximity may partially contribute to the strength of observed associations and limit the extent to which effects can be interpreted as fully independent or mechanistically distinct. Thus, the present findings are best understood as delineating structured relationships within a shared attitudinal and belief‐based domain, rather than as evidence of isolated causal predictors. In other words, our findings are relevant for understanding how clusters of beliefs and orientations jointly shape acceptance of AI‐based DMHIs.

Moreover, although the UTAUT‐AI‐DMHI has recently been validated in the US and UK samples, it remains unclear if the same scale psychometrics apply across countries or cultures. Other similar self‐report scales have since been developed in other countries that might be important for further validation across borders. For example, the Artificial Intelligence in Psychotherapy Scale (AIPS) reported by Bilge et al. (2025) has recently been developed in a Turkish sample to assess these attitudes.

Furthermore, participants' understanding of chatbot and avatar interventions was based on brief visual examples rather than direct experience, which may have limited ecological validity (e.g., Aafjes‐van Doorn 2025). Future studies should include interactive demonstrations to capture more realistic acceptance levels (as was done for avatar‐based healthcare tools by Moriuchi 2025).

Lastly, given that the field is developing at a rapid pace, it is possible that these reported acceptance levels are outdated by the time this manuscript is published. Further follow‐up studies and replications are warranted to track the change in acceptability among these three groups, while individuals become more familiar with AI technology more generally and while avatars become more realistic.

### 3.4 Clinical Implications

Although the cross‐sectional design precludes conclusions about causal mechanisms, our findings may inform hypothesis generation, clinical reflection and future intervention development. Specifically, the acceptability of DMHIs is important for clinical implementation and training, for tool design and engagement and for ethical considerations.

Given the greater scepticism towards AI‐based tools among clinicians, targeted efforts are needed to support their familiarity and confidence. Training programs, exposure to evidence‐based efficacy data and opportunities to trial AI‐based systems may help clinicians build trust in their usability, reliability and therapeutic potential. Integrating psychoeducation about AI applications in healthcare, highlighting real‐world examples where AI complements (rather than replaces) human care and involving clinicians in the co‐design of DMHI tools could improve professional acceptance and reduce concerns about usability and therapeutic effectiveness.

Community participants, and to a lesser extent patients, perceived greater potential for human connection when interacting with AI chatbots and avatars than did clinicians. This suggests that developers should focus on relational and emotional design features to foster engagement. Empathetic language models, personalised avatars and emotionally responsive interfaces may strengthen the perceived sense of rapport and improve adherence. These features should also address one of the major challenges in DMHI use: maintaining user engagement over time (Aafjes‐van Doorn et al. 2025; Beg and Verma 2025). Additionally, our moderation findings on age suggest that developers might consider age‐sensitive design, such as adaptive interfaces or tailored onboarding tutorials, to meet the differing expectations and experiences of various age groups.

The consistently high acceptance of teletherapy, particularly among patients, indicates that hybrid care models combining teletherapy with AI‐based components may be both feasible and desirable. AI chatbots or avatars could supplement human‐delivered care by providing between‐session support, psychoeducation or crisis response tools, extending the reach of clinicians while preserving therapeutic relationships. Emerging evidence, such as the ‘Friend’ chatbot providing immediate crisis support (Spytska 2025) and avatar‐assisted telecare improving adherence and clinical outcomes (Winkler et al. 2025), supports the potential of these integrated models.

Beyond technical limitations, such as model memory and bias, the use of AI in mental health care raises substantial ethical concerns regarding privacy, autonomy and fairness. As noted by Walsh et al. (2020), systems that rely on sensitive data must carefully balance improved care with the protection of patient rights. Transparent design, mitigation of algorithmic bias and maintaining human oversight are essential to preserving trust (Beg et al. 2025; Li et al. 2023, 2024). Without rigorous ethical governance, AI‐based systems risk unintended harms such as misdiagnosis or erosion of the therapeutic alliance. These challenges parallel broader concerns in precision psychiatry (Fusar‐Poli et al. 2022), where AI tools must enhance rather than replace the nuanced understanding that human therapists bring to care.

Finally, while AI‐based DMHIs can improve accessibility and affordability, barriers remain. Teletherapy services often remain expensive and limited by clinician availability, while AI chatbots and avatars, though generally cheaper, are frequently locked behind paywalls or language restrictions. This uneven access means that many potential patients may turn to general‐purpose large language models such as ChatGPT or DeepSeek for mental health support (Giray 2025), despite these not being designed or validated for therapeutic use. Future research should therefore examine both the acceptability and ethical implications of such informal AI support systems.

## 4 Conclusion

Growing demand for broadly accessible mental health care, together with the rapid development of new AI‐based technologies, triggers discussions about the feasibility of AI‐based DMHI. Our study suggests that community participants were most optimistic about AI‐based mental health tools, whereas clinicians expressed greater scepticism, particularly regarding usability. In our sample, general attitudes towards AI were strongly associated with acceptance, more than demographic or professional factors. As exposure to AI tools grows, collaboration among clinicians, patients, developers and researchers will be essential to ensure that these technologies enhance, rather than replace, human care.

## Funding

This study was supported by the Dr. Nancy Hollander Research Fund (VB).

## Ethics Statement

The study followed the US Federal Policy for the Protection of Human Subjects ethical guidelines and was undertaken with the consent of each participant. The study was reviewed and deemed exempt by Yeshiva University's Institutional Review Board (#1‐1758995‐1).

## Conflicts of Interest

The authors declare no conflicts of interest.

## Data Availability

The data that support the findings of this study are available from the corresponding author upon reasonable request.

## Endnotes

^1^
Chatbots For Mental Health and Therapy Global Market Report 2025—By Component (Software As A Service (SaaS), Other Components), By Technology (Machine Learning And Deep Learning, Natural Language Processing, Other Technologies), By Application (Conversational Interfaces, Behavioral Pattern Recognition, Other Applications) – Market Size, Trends, And Global Forecast 2025–2034.
